# Scaling up noncommunicable disease care in a resource-limited context: lessons learned and implications for policy

**DOI:** 10.1186/s12913-024-11328-x

**Published:** 2024-07-25

**Authors:** Yoseph Mamo, Mirchaye Mekoro, David I. W. Phillips, Andrew Mortimore

**Affiliations:** 1 Tropical Health Education Trust, Addis Ababa, Ethiopia; 2 Health Poverty Action, Addis Ababa, Ethiopia; 3 MRC Lifecourse Epidemiology Centre, Southampton General Hospital, University of Southampton, Tremona Road, SO16 6YD Southampton, UK; 4 Academic Unit of Primary Care, Population Sciences and Medical Education, Faculty of Medicine, University of Southampton, Southampton, UK

**Keywords:** Ethiopia, Non-communicable disease, Implementation research, Comprehensive care, Decentralisation

## Abstract

**Background:**

Although primary care models for the care of common non-communicable diseases (NCD) have been developed in sub-Saharan Africa, few have described an integrated, decentralized approach at the community level. We report the results of a four-year, Ethiopian project to expand this model of NCD care to 15 primary hospitals and 45 health centres encompassing a wide geographical spread and serving a population of approximately 7.5 million people.

**Methods:**

Following baseline assessment of the 60 sites, 30 master trainers were used to cascade train a total of 621 health workers in the diagnosis, management and health education of the major common NCDs identified in a scoping review (hypertension, diabetes, chronic respiratory disease and epilepsy). Pre- and post-training assessments and regular mentoring visits were carried out to assess progress and remedy supply or equipment and medicines shortages and establish reporting systems. The project was accompanied by a series of community engagement activities to raise awareness and improve health seeking behaviour.

**Results:**

A total of 643,296 people were screened for hypertension and diabetes leading to a new diagnosis in 24,313 who were started on treatment. Significant numbers of new cases of respiratory disease (3,986) and epilepsy (1,925) were also started on treatment. Mortality rates were low except among patients with hypertension in the rural health centres where 311 (10.2%) died during the project. Loss to follow up (LTFU), defined as failure to attend clinic for > 6 months despite reminders, was low in the hospitals but represented a significant problem in the urban and rural health centres with up to 20 to 30% of patients with hypertension or diabetes absenting from treatment by the end of the project. Estimates of the population disease burden enrolled within the project, however, were disappointing; asthma (0.49%), hypertension (1.7%), epilepsy (3.3%) and diabetes (3.4%).

**Conclusion:**

This project demonstrates the feasibility of scaling up integrated NCD services in a variety of locations, with fairly modest costs and a methodology that is replicable and sustainable. However, the relatively small gain in the detection and treatment of common NCDs highlights the huge challenge in making NCD services available to all.

**Supplementary Information:**

The online version contains supplementary material available at 10.1186/s12913-024-11328-x.

## Summary points

There is a large body of literature recommending decentralisation of noncommunicable disease (NCD) care, but extremely few “real-world” examples at scale. Those that do are largely examples of NCD care limited to single diseases and in similar geographical or cultural settings. This project provides screening, enrolment and clinical outcomes data for fully integrated, multi-level NCD clinics across a wide geographical area in Africa’s second most populous nation.

It is one of the first examples of scaled-up comprehensive care for all-comers with chronic noninfectious disease in rural and urban Ethiopia.

The extra costs and effort involved in staff training, mentoring and community engagement are not inconsiderable for a modest gain in the detection and treatment of common NCDs.

Its major limitation is that it is a “real-world” intervention and observational cohort, studied over a period constrained by a global pandemic and internal civil conflict. It uses routinely collected clinical data, limiting the ability to fully evaluate all relevant clinical outcomes.

**Supplementary Information:**

The online version contains supplementary material available at 10.1186/s12913-024-11328-x.

## Introduction

Non-communicable diseases (NCDs) are fast becoming the major cause of death and disability in many resource-limited countries especially in sub-Saharan Africa. In Ethiopia, NCDs together with injuries currently account for 44% of the total annual mortality [1] with cardiovascular/respiratory disease and diabetes being the major causes [2]. Yet the country remains among the world’s lowest income countries with a *per capita* gross national income of $960 and 24% of the population living below the poverty line (US$ 1.90 per day) with correspondingly low *per capita* health expenditure [3]. In addressing this problem the World Health Organization (WHO) has emphasized the importance of strengthening primary care systems and integrating cost-effective NCD interventions [4]. However, in Ethiopia as with many other resource-limited countries in sub-Saharan Africa, these systems remain poorly developed, particularly in the rural areas where most of the population live.

Primary care in Ethiopia is delivered through a system of small primary (district) hospitals, health centres and health posts. Although available to most of the population, their ability to provide effective NCD services has been limited by a focus on acute care delivery (vaccinations, communicable diseases, and maternal and child health), deficiencies of appropriate equipment and medicines together with the lack of expertise. As a result, NCD care has been restricted to the secondary or tertiary hospitals found in the larger towns and cities. Over 20 years ago we developed a decentralized model for NCD care in Ethiopia, concentrating on locally prevalent conditions [5]. This was based on a group of 17 rural health centres situated around the University Hospitals of Gondar and Jimma, 750 km northwest and 330 km southwest of the capital, Addis Ababa, respectively. Nurses in the health centres through in-service training and support were enabled to effectively diagnose and initiate treatment in uncomplicated cases [6, 7].

In 2014 the Ethiopian Ministry of Health (MoH) adopted this model and turned to partners to help scale up NCD care as part of its national strategic action plan, which emphasizes the need to strengthen and reorientate health systems to address prevention and control of NCDs through people-centred primary care and universal health coverage [8]. We report the results of a four-year project to expand this model of NCD care to primary hospitals and health centres in a variety of different locations with a wide geographical spread within Ethiopia.

## Methods/description of project

### Organizational context

**Fig. 1:**
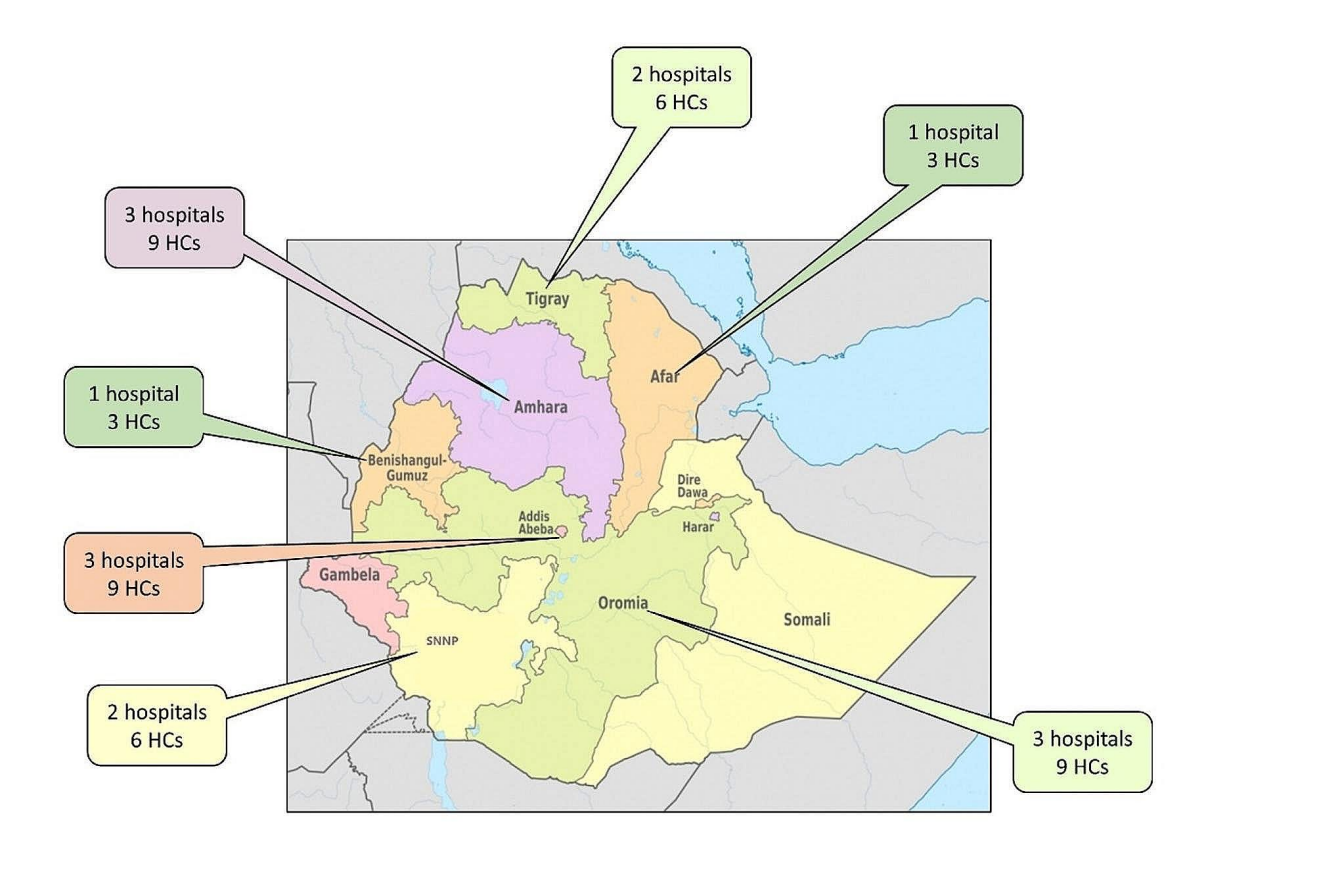
Location of initial project sites

Two NGOs, The Tropical Health Education Trust (THET) and Health Poverty Action (HPA), with extensive experience in decentralizing NCD care and primary health care interventions including community development, respectively, partnered with the Ethiopian MoH in a Novartis Social Business-funded project in 15 hospitals and 45 health centres (Fig. 1) with the aim of increasing access to services for NCDs in six Ethiopian regions (Amhara, Tigray, Oromia, SNNPR, Benshangul-Gumuz and Afar) and one city administration (Addis Ababa, the capital city). Because of the outbreak of civil conflict, the eight original project sites in Tigray became inaccessible and were replaced with new sites in Benshangul and Afar. Of the 15 hospitals, three were general hospitals in Addis Ababa and 12 primary hospitals across the selected regions, while among the 45 health centres, 14 were urban and 31 rural. Essential NCD medicines were supplied through the MoH and dispensed at low cost or free for those on very low incomes.

### Baseline evaluation

Comprehensive assessments were carried out in all 60 project sites using a modified version of the WHO Service Availability and Readiness Assessment (SARA) tool [9]. Structured interviews of hospital or health centre staff, reviews of medical records and general observation of the resources available provided information on the human resources available, the current patient throughput, laboratory facilities and pharmaceutical supplies relevant to NCDs.

The main findings are summarised in Table 1. Total health worker staffing ratios were higher in the urban than the rural facilities and although these figures are comparable with other sub-Saharan African countries, [10] they are well below the WHO guideline of 4.45/1,000 required for universal health coverage. There were no doctors below the primary hospital level. All the urban centres and almost all the health centres had access to water and power supplies although periodic interruptions were common in each region. All had some form of power back up. The urban hospitals served large populations with their catchment area overlapping somewhat with other providers. In contrast, there was enormous variation in the population covered by the health centres which ranged from as little as 2,000 to 92,000 in some locations depending on the area and population density. While the hospitals were well-equipped with quality assured sphygmomanometers, glucose, HbA1c, renal and liver function tests together with treatment protocols and appropriate medicines, these were severely lacking in the health centres. Among the 45 health centres surveyed although over 80% said they had access to sphygmomanometers most of these were substandard and poorly maintained. Less than a quarter had glucometers, reporting that sticks were often hard to obtain and available investigations were often limited to a blood count and dipstick urinalysis. None had peak flow meters. Less than one third had staff with any NCD training and 90% of health centres had no NCD teaching materials.

**Table 1:** Baseline data from the project hospitals and health centres

	Nature of health facility			
	Addis Ababa hospitals	Primary hospitals	Urban health centres	Rural health centres
Total No.	3	12	14	31
No. of linked health posts	NA	360	20	178
Catchment population*	900,000	6,576,423	628,504	981,179
Total Staff	1,230	870	692	569
Doctors	141	127	0	0
Nurses	953	569	380	308
Health Officers	44	24	159	69
Pharmacists	89	118	110	70
IT staff	3	32	43	36
Overall staffing ratio (per 1,000 population)	1.36	0.13	1.1	0.58
Distance to nearest referral hospital (mean in km)	5	171	6	43

*In some instances, the catchment populations of hospitals overlap with the catchment populations of health centres

### Project design

#### Training

A system of cascade training was developed for the project. Initially, 30 general practitioner Master Trainers (MTs) were recruited from the 15 hospitals in the project with the aim that they would be leaders of change in improving NCD services in their respective hospitals and satellite health centres. During a 10-day residential course, training was delivered to cover the major common NCDs identified in a scoping review (hypertension, diabetes, chronic respiratory diseases and epilepsy) [1], the use of the Practical Approach to Care Kit (PACK) algorithms [11], together with instruction on training methods, particularly adult teaching and mentoring skills. Pre- and post-test evaluations were carried out before and after each topic. The MTs subsequently delivered a four-day training course to their respective catchment health centre staff using both didactic and interactive teaching methods. Again, daily pre- and post-training assessments were used to evaluate the knowledge and skills developed. A total of 621 health workers were trained to be responsible for patient screening, diagnosis, treatment and health education, including training for Health Extension Workers (HEWs). In the second year an additional ‘gap-filling’ training was arranged for health workers to fill vacancies created by health workers who had been trained but left their institutions or changed their job role. Following their return to their health facility, they were encouraged to start seeing and managing patients with NCDs, seeking advice from senior staff in the health centres or primary hospitals if necessary. Diagnostic criteria and management protocols for NCDs together with their risk factors, e.g. smoking, alcohol and obesity, were according to PACK guidelines. Supplementary Table 1 lists the drug treatments which were available in the health centres and hospitals. Patients were usually prescribed one month’s supply of medicines which cost approximately 2–4 US$ /month, although a waiver system in Ethiopia permits access to free health care for those most impoverished.

#### NCD equipment and data collection

As many of the health centres did not have access to the equipment required for the diagnosis and management of NCDs, basic equipment including accurate weighing scales, quality assured sphygmomanometers (two per health centre) and glucometers (one per health centre with sticks) were provided. Both the health centres and hospitals received the MoH data collection tools which included patient registers (identified by name and unique ID numbers), tally sheets, intake forms and follow-up forms with reporting formats for each NCD covered by the project training. Data from the clinics were compiled into monthly reporting formats, sent to the THET office in Addis Ababa and entered into spread sheets which were then analysed by the monitoring and evaluation team leader.

#### Mentoring and supervision

Regular visits to both hospitals and health centres were carried out by a team consisting of members of the project team, MTs and local health office staff to discuss overall progress and identify any problems, for example drug supplies or equipment shortages. The MTs also visited the health centres regularly to give them in-service training using available cases or PACK to remedy gaps in diagnostic or patient communication skills. In addition, there were regular catchment area meetings involving local staff and annual stakeholder meetings in Addis Ababa for MoH NCD directorate staff, project staff, local health office staff, medical directors and facility heads. This involved a total of 458 mentoring visits, equivalent to 1,374 days over the 4½ years of the project with an emphasis on staff motivation and a cycle of continuous quality improvement.

#### Community engagement

During the implementation of the project, a series of community engagement activities were held to raise awareness about NCDs aimed at improving health-seeking behaviour. A total of 1,045,302 people were reached through events such as screening sessions, car-free days, market days and other gatherings in the intervention areas. In addition, a total of 6,714 religious and community leaders were engaged in a series of dialogues to help address the social norms and taboos which hinder people from accessing health services. Finally, specially designed radio messaging was broadcast to reach an estimated audience of more than one million people, providing information on NCDs, their risk factors and available treatment in nearby health centres.

#### Screening for NCDs

The local health staff in the project areas were encouraged to screen people opportunistically for NCDs (hypertension and diabetes) in triage/outpatient rooms in health centres. Screening for hypertension was carried out in all patients over 18 years and for diabetes in > 40 year olds or those with hypertension. Other strategies used included carrying out open-air campaigns on car-free days, at marketplaces and the entrance to government offices. For the open-air events a small tent was erected with loudspeakers broadcasting music and announcements together with the distribution of leaflets. People with raised blood pressure or blood sugar were given a referral slip for further tests at primary hospitals or health centres. HEWs were trained to recognize symptoms of epilepsy or asthma and arrange appropriate referral.

## Results

### Project activity

Between November 2018 and March 2023, 643,296 people were screened for hypertension and diabetes leading to a new diagnosis in 24,313 who were started on treatment. Table 2 shows the total numbers of new NCD cases registered during the project according to diagnosis and type of health facility. Large numbers were screened for hypertension in all locations with the highest detection rate in Addis Ababa (7.3%) and among the primary hospitals (5.1%). Detection rates in the health centres were much lower (1.6% and 2.5% in the urban and rural health centres respectively). Because of the age restriction fewer people were screened for diabetes; nevertheless significant numbers of new cases were detected. Highest detection rates were reported in the Addis Ababa and primary hospitals (59.6% and 14.9% respectively) with much lower rates in the health centres (3.4% and 1.1% in urban and rural health centres respectively). For respiratory disease and epilepsy there were no specific efforts to find patients, yet significant numbers were enrolled in all types of facility. Reported mortality rates were low with the exception of hypertension in the rural health centres where 311 (10.2%) died over the course of the project. Loss to follow up (LTFU), defined as failure to attend clinic for > 6 months despite reminders, was low in the hospitals but represented a significant problem in the urban and rural health centres with up to 20 to 30% of patients with hypertension or diabetes absenting from treatment by the end of the project.

**Table 2:** Numbers screened for hypertension and diabetes together with numbers of NCD cases enrolled to care during the project according to diagnosis and type of health facility. Reported all-cause mortality and loss to follow up (LTFU) over the duration of the study are also shown

	Type of health facility			
	Addis Ababa hospitals	Primary hospitals	Urban health centres	Rural health centres
Number of facilities	3	12	14	31
Hypertension				
Screened	66,880	55,467	293,235	123,212
Enrolled (%)	4,894(7.3)	2,813(5.1)	4,815(1.6)	3,045(2.5)
Died	0	8	5	311
LTFU	3	88	837	251
Diabetes				
Screened	7,447	14,008	58,288	24,759
Enrolled (%)	4,441 (59.6)	2,083(14.8)	1,953(3.4)	269(1.1)
Died	0	5	7	6
LTFU	39	46	439	77
Respiratory				
Enrolled	881	360	1,950	795
Died	8	0	15	2
LTFU	0	52	3	49
Epilepsy				
Enrolled	480	639	499	307
Died	0	32	0	15
LTFU	0	2	1	15

### Project impact

To evaluate the project’s impact on the overall burden of disease in the population, we compared the numbers of patients enrolled with estimates of the potential total numbers of cases for each NCD, derived from published population prevalence data, according to the type of health facility (Table 3). For all NCDs only a small proportion of the potential number of cases in the population had been enrolled within the project. Asthma and hypertension had the lowest overall detection rates (0.49% and 1.7% respectively) while epilepsy (3.3%) and diabetes (3.4%) had the highest. However, there was wide variability between different types of facility. Detection rates were highest in the Addis Ababa hospitals and were lowest in the primary hospitals while urban health centres achieved higher rates than the rural health centres. The reported levels of control after 6 months treatment for hypertension (defined as achieved blood pressure < 140/90 mmHg) or diabetes (random blood sugar < 180 mg% or fasting blood sugar < 130 mg%) in the quarterly returns were consistently greater than 85%.

**Table 3:** Numbers of patients diagnosed as a proportion of the total estimated caseload for each of the major diagnoses, according to the type of health facility

	Nature of health facility			
	Addis Ababa hospitals	Primary Hospitals	Urban health centres	Rural health centres
Number	3	12	14	31
Catchment population	900,000	6,576,423	628,504	981,179
Hypertension				
Estimated prevalence^1^ (%)	15	15	15	7
Predicted caseload	67,500	493,232	47,138	34,341
Case detection (%)	4,894 (7.3)	2,813(0.6)	4,815(10.2)	3,045(8.9)
Diabetes				
Estimated prevalence^1^ (%)	6	6	6	3
Predicted caseload	27,000	197,293	18,855	14,718
Case detection (%)	4,441 (16.4)	2,083(1.1)	1,953(10.4)	269(1.8)
Asthma				
Estimated prevalence^2^ (%)	9	9	9	9
Predicted caseload	81,000	591,878	56,565	88,306
Case detection (%)	881(1.1)	360(0.06)	1,950(3.4)	795(0.9)
Epilepsy				
Estimated prevalence^3^ (%)	0.64	0.64	0.64	0.64
Predicted caseload	5,760	42,089	4,022	6,280
Case detection (%)	480(8.3)	639(1.5)	499(12.4)	307(4.9)

^1^ref [17] ^2^ref [22] ^3^ref [23]

Predicted caseload based on estimated adult populations for hypertension and diabetes and total populations for asthma and epilepsy

### Finance

Table 4 shows the estimated annual costs of the project (provided by the donor) together with an estimate of the cost per patient enrolled. Salaries for the Addis Ababa-based staff were a major component as were travel costs given the widely dispersed location of many of the project sites. However, community engagement proved to be the most expensive overall cost.

**Table 4:** Project costs

	Project cost per year (US$)
Addis-based project staff	50,469
Travel and training*	75,407
Equipment*	16,099
Community engagement	86.702
Total cost	228,677
Cost per patient enrolled	10.5

*Annualised costs

#### Staff turnover

Rapid turnover of staff was a major problem. Of the original 30 mentors trained, only 10 were still in place by the end of the project. To replace these a further 17 new mentors were trained. At health centre level almost 80% had moved on by the end of the project and we needed to re-train mentors for almost all sites. HEWs were least affected by turnover and all 610 trained remained in place for the duration of the project.

#### Drug and equipment availability

Essential medicines for hypertension, diabetes, asthma and epilepsy were available in almost all hospitals at the start of the project although sustaining adequate supplies proved to be a major problem because of difficulties with the national supply chain system. The situation was different in the health centres. At the start of the project NCD medicines and diagnostic equipment were patchily available in some of the centres. Midway through the project 32 of the health centres answered a short questionnaire asking about availability of essential NCD medicines. Hypertensives were available in all but four centres (23 had diuretics, 22 ACE inhibitors and 28 calcium channel blockers); diabetes medications in 25 (biguanides in 19 and sulphonylureas in 22); anticonvulsants in 20 and asthma inhalers in just 14. At the end of the project a glucometer and two sphygmomanometers/stethoscopes were present in all the NCD clinics. However, supplying enough test strips for glucometers proved to be difficult.

## Discussion

We report the outcome of a large-scale intervention to deliver NCD services for major common diseases at the community-level where hitherto there was little or no provision. The intervention was embedded within the existing Ethiopian health service, using the existing staff and infrastructure. The project sites included large hospitals in the capital city, Addis Ababa, primary hospitals and both urban and rural health centres and for reasons of equality had a wide geographical coverage (Fig. 1) encompassing a population of approximately 7.5 million people (allowing for overlap of the catchment between large hospitals and between primary hospitals and their satellite health centres). The project was underpinned by close relationships with the MoH at national, regional and district (woreda) level and co-designed with the MoH, and the core activities were delivered by their personnel. Every aspect of set-up was tested and refined through processes of feedback, reflection, and re-design. The tools and methods jointly developed continue to be used and are part of the legacy of the project. Although a number of primary health care models of NCD care have been developed in sub-Saharan Africa, few have described an integrated, decentralized approach at the community level. A recent review of NCD and mental health interventions identified 188 studies of which only 29 were conducted in low-income countries [12]. Of these, just 11 were at the community or health centre level, and most focussed on a single disease or were small-scale pilot studies. Of the four comparable studies two were from Malawi [13, 14], one from the Cameroon [15] and data from our previous work in Ethiopia [5].

Assessing the impact of the project on NCD services and the burden of disease in the community is more difficult. The data from the health centres suggests that a large number of nurses and health officers had been successfully trained and that the availability of basic equipment had substantially increased as a result of the project. The screening process was particularly successful with large numbers being assessed for hypertension or diabetes. Table 2 also shows that the screening process for hypertension had a higher yield in the hospital-based projects (7.3% and 5.1%) than in the health centres (1.6% and 2.5%) which probably reflects the higher prevalence rates in the screened populations. However, prevalences in both urban and rural areas are lower than expected. Possible explanations include the known widespread suspicion and poor utilisation of NCD services in the Ethiopian population due to a preference for traditional treatments [16] and differences between diagnostic criteria used in the STEPS prevalence surveys [17] and those used in national treatment guidelines.

In a similar way, diabetes was much more likely to be detected among the hospitals than the health centres. The very high prevalence recorded in the Addis Abba hospitals is clearly an outlier and may have included patients with pre-existing diabetes. Although people being screened were asked whether they already had the condition, the treatment of patients is often erratic and difficulties in sourcing treatment may have encouraged self re-referral to obtain low-cost hypoglycaemic medications.

The health facility staff were also trained to manage epilepsy and respiratory disease. Although these conditions were not part of the systematic population screening programme, significant numbers presented to both hospitals and health centres and were enrolled for treatment as shown in Table 2.

Reported all-cause mortality rates were low (Table 2) with the exception of rural patients with hypertension which is unexplained. The possibilities include inadequate treatment (we do not know whether those that died had defaulted from clinical follow-up), the possible impact of COVID-19 (though it is not clear why this should be worse in rural areas) or other, unrelated causes of death.

Most people attending clinics were adequately controlled although our data are not complete enough to make definitive statements and follow-up will be needed to see whether this is maintained over the longer term. We have previously shown that staff in rural clinics in Ethiopia are well able to manage hypertension using simple regimes [6]. However, despite intensive outreach and engagement, uptake was low and retention on treatment somewhat disappointing (Table 3) although the duration of the project may have been too short to build up the level of community engagement and trust in the clinics and forms of NCD management required to make a substantial impact on the disease burden. Previous work in Ethiopia has demonstrated the numerous barriers to treatment, including access/distance, consistency of drug availability, cost (out-of-pocket expense and time) and cultural beliefs and practices related to health and healing [16].

One problem that became evident during the project was competition with other vertical health programmes. This was particularly evident in the more remote, lower-level care settings, where the NCD project was introduced alongside other, often better-resourced programmes (e.g. antenatal care; water, sanitation and hygiene (WASH); under-fives; tuberculosis (TB) and HIV/AIDS). Despite the expansion of all cadres of health staff in Ethiopia, multiple programmes are delivered by the same, hard-pressed and resource-constrained staff in rural areas. Related to this was the problems many patients reported in navigating the NCD care system resulting in confusion, mixed messaging, duplication of tests and an inefficient use of resources. It is clear that more attention needs to be given to developing the continuum of care for NCDs at all levels: supporting patients throughout their care journey from health promotion, prevention, diagnosis, treatment, to rehabilitation, and/or palliative care. Integrating NCD services with other services such as HIV/AIDS, TB etc. should be considered, as has been the case in Kenya, Uganda, Zambia, Malawi and elsewhere [18–20].

Few, if any studies report the financial implications of projects such as the one we describe. While project funding is complex and dependent on the scale and geographic reach as well as the economic environment, our data (Table 4) suggest that while the per-patient costs are fairly low, the costs are likely to be considerable especially if the ongoing costs of medicines are included. NCD patients tend to be multiply disadvantaged compared to those needing episodic treatment for acute illnesses or who are within funded programmes such as HIV/AIDs and TB. Their need for continuous treatment and the concept of a disease that cannot be cured and needs lifelong medication places a long-term financial burden on the family, is counter-cultural and is a message at variance with those coming from other traditional systems of healing praxis. Although community-based insurance schemes were introduced in the country for poor rural patients (in 2011), even a co-payment system is likely to be a prohibitive drain on household finances in the long-term. Furthermore, enthusiasm to take part will be dampened for those not incurring healthcare costs, and the scheme will be unable to generate significant funds if only those with NCDs contribute in the long term.

One of the major problems encountered in the project was ensuring accurate and consistent completion of the paper-based record system which depended on patient registers, tally sheets and intake forms for each NCD. The overly detailed registration forms were burdensome and led to incomplete and inaccurate recording which will have affected data quality in this report which, consequently, is likely to have underestimated the throughput of patients and loss to follow-up. Although during the four years of the project a number of improvements and revisions were made at central level, improved record keeping is still needed. One solution may be the introduction of electronic medical records and linked IT systems which can facilitate clinical decision-making and enhance the ability to monitor outcomes, as has been shown in Malawi and elsewhere [21]. However, a cultural shift will be needed to promote the value of data in understanding what is being achieved and in driving up the quality and effectiveness of services.

The availability of affordable medicines is essential for the running of NCD clinics and patient compliance [16] and, although essential medicines were provided by the MoH, at the local level supplies were not always available. In a survey of 32 health centres, while the majority had available antihypertensive and diabetes medicines a significant minority did not. Even fewer centres reported the availability of medicines for epilepsy or inhalers for asthma. Most of the health centres reported that the main reason for the poor availability was a slow-to-respond supply chain mechanism which lacked efficient and effective communications between its various levels and components.

Staff turnover also proved to be a problem and required the identification of replacement trainers and mentors for almost for all sites. Loss of staff was most evident at the hospital and health centres level. By contrast all the HEWs trained during the project were retained for its duration. The likely reasons for this are the greater alternative job opportunities and financial advancement for urban-based staff coupled with the high workload and poor working environments.

Finally, the arrival of COVID-19 in Ethiopia and the outbreak of civil conflict in Tigray had a significant effect on the project as travel was restricted, and it became more difficult for patients to attend hospital or health centre clinics. Despite this, mentoring and supervision of the clinics continued utilising IT solutions while clinic staff used mobile phones to keep in contact and ensure the follow-up of NCD patients.

In summary, this project has demonstrated the feasibility of scaling up integrated NCD services in a variety of different locations, with fairly modest costs, and a methodology that is replicable and we believe sustainable as it was based on our long-running NCD decentralisation projects in Gondar and Jimma [5]. An important aspect was the development of training and supervisory methods in partnership with the Federal Ministry of Health and embedding these within existing district health administration structures who have now taken on responsibility for the post-project supervision. In addition the supply of basic NCD medicines and diagnostics are now included in the list of essentials for health centres with purchase permissions which did not exist before.

It is difficult to be certain which aspects of the project were most successful. It is likely that the combination of training, equipment provision and ensuring the supply of medicines are critical but the screening programmes for hypertension and diabetes greatly boosted the numbers of patients enrolled. While taking advantage of existing staff and infrastructure is important, the extra costs and effort involved in staff training, mentoring and community engagement are not inconsiderable for a modest gain in the detection and treatment of common NCDs. In addition, given that there are over 3,500 health centres in Ethiopia, the process of making NCD services available to all represents a huge challenge. This will need to be carried out in an evidence-based way, addressing known challenges and adapting to new ones. For the effort and investment to have the greatest impact, improving the uptake and reducing the numbers lost to treatment must be a key focus. This may include the use of IT technologies and telecare systems if these can be operated cheaply and at scale, together with further decentralisation of care so that HEWs and communities play a much greater part in health education, recognition, control and management of NCDs.

### Electronic supplementary material

Below is the link to the electronic supplementary material.

Supplementary Material 1

## Data Availability

While the data used in this study are not publicly available, summary data are available upon request to the corresponding author.

## Abbreviations

ACE: Angiotensin converting enzyme
HEW: Health extension workers
IT: Information technology
LTFU: lost to follow up
NCD: non-communicable disease
MT: Master Trainers
MoH: Ministry of Health
PACK: Practical approach to care kit
SARA: Service availability and assessment tool
SNNPR: Southern Nations, Nationalities, and Peoples’ Region
THET: Tropical Health Education Trust
WHO: World Health Organisation
