# Supplementary material for: Scaling up noncommunicable disease care in a resource-limited context: lessons learned and implications for policy
Source: BMC Health Serv Res. 2024 Jul 25;24:847. doi: 10.1186/s12913-024-11328-x (PMC11282768; doi:10.1186/s12913-024-11328-x)
Supplement: Supplementary file 1 — Supplementary Material 1 [file 12913_2024_11328_MOESM1_ESM.docx]

Supplementary Table 1: Available treatments for NCDs.

| NCD category | First line drugs | Additional drugs* | Available in hospitals only |
| --- | --- | --- | --- |
| Hypertension | Hydrochlorthiazide, amlodipine, nifedipine | Enalapril | Atenolol, hydralazine |
| Diabetes | Metformin, glibenclamide | Glyburide | Insulin (soluble, intermediate and mixed) |
| Epilepsy | Phenobarbitone | Phenytoin, carbamazepine, sodium valproate |  |
| Asthma | Salbutamol/aminophylline/theophylline tablets | Salbutamol/beclomethasone inhaler |  |

*Sometimes available
